# A case-based approach to teaching epidemic and pandemic-related global health diplomacy and security in African countries

**DOI:** 10.1186/s12992-022-00849-x

**Published:** 2022-05-26

**Authors:** Sebastian Kevany, Shayanne Martin, Mike Reid

**Affiliations:** 1 Asia-Pacific Center for Security Studies, 2058 Maluhia Road, Honolulu, HI 96815 USA; 2 University of California, San Francisco, USA

**Keywords:** Global health diplomacy, Global health security

## Abstract

A challenging concept to teach, few combined courses on epidemic-related global health diplomacy and security exist, and no known courses are currently available that have been exclusively designed for African nationals. In response, the University of California, San Francisco’s Center for Global Health Delivery, Diplomacy and Economics (CGHDDE) developed and delivered a workshop for LMIC learners to better understand how politics, policy, finance, governance and security coalesce to influence global health goals and outcomes.

Through international relationships, discussions and negotiations, global health diplomacy and security efforts can promote and enable rapid, coordinated responses to pressing global health issues, including the current and immediate ones posed by viral pandemics [1]. Contemporary concerns exemplify the need for such skills worldwide; however, in the context of the ongoing shift towards domestic (also known as ‘local’ or ‘country’) ownership of health programs, policy makers, researchers and public health professionals from African countries continue to represent an unmet need for health diplomacy and security skills. Moreover, there is today an urgent and increasing need to ensure local capacity for health security and associated surveillance efforts is prioritized in the Global South.

A challenging concept to teach, few combined courses on epidemic-related global health diplomacy and security exist, and no known courses are currently available that have been exclusively designed for African nationals. In response, the University of California, San Francisco’s Center for Global Health Delivery, Diplomacy and Economics (CGHDDE) [2] developed and delivered a workshop for African learners to better understand how politics, policy, finance, governance and security coalesce to influence global health goals and outcomes.

Design was soon followed by delivery: in August 2019, the authors delivered a satellite half-day workshop in association with the Third Annual AFREhealth (African Forum for Research and Education in Health) Symposium in Lagos, Nigeria. The composition of the teaching team was focused on CGHDEE global health diplomacy and security expertise, though in future (and in line with the decolonization of global health education) a Global South instructor would also be valuable.

Using a problem-based learning approach, the 100+ participants were presented with introductions to key global health diplomacy and security concepts of intervention adaptability [3]; sustainability of services under donor transition [4]; ethics and economics in resource allocation decisions [5]; and a range of other topics related to epidemic and infectious disease control. Participants representing more than a dozen different African countries (selected via expressions of interest and global health networks) and with backgrounds in government, research, and non-governmental organizations were then invited to discuss, in small groups, case studies that demonstrated the role of diplomacy in responding to global health security and epidemic control challenges.

Comparison of pre- and post-workshop surveys demonstrated self-reported increased understanding of the concepts of ethics and global health service delivery; global health security; global health stakeholders; and donor financing transition in a group with little prior reported formal training in the field of global health diplomacy. Participants most commonly reported benefits from the interactive nature of the group casework, although both the trainers and participants noted more time was needed for case study discussion. These findings suggest that there is a significant unmet demand for more widespread training in the concepts of both health diplomacy and security – in particular, how the two realms interact in epidemic or pandemic contexts. In many cases, particularly when military involvement is required (as in the 2014 West African Ebola outbreak) [6, 7], health security and infectious disease control efforts require intensive diplomatic and political efforts for success – not least to ensure that health gains are not offset by international relations losses.

In order to enable related conceptual understanding, in preparing the didactic content of the workshop, our team deliberately moved away from the concept of “donor” and “recipient” countries, concepts which may too easily lose relevance and immediacy in the health security context, in which it is vital that all countries, regardless of development status, adopt equally high standards. However, case study discussions also revealed the challenges of re-orientating global health diplomacy and security concepts from Global North to Global South learners in this regard; too often, health security and diplomacy was found to be perceived as protecting the economic and health interests of more affluent countries, at the expense of the Global South.

Such shortcomings were perhaps inevitable: to our knowledge, this was the first global health diplomacy and security training (and which is adaptable to virtual settings) developed specifically for African learners. While our case-based approach was successful, our experience also demonstrated that more time is needed to allow learners to walk through real-world applications or devise solutions that would be acceptable to local, national and international global health stakeholders. Thus, despite an effort to re-position global health diplomacy and security in terms of donor transition and African ownership, such mixed reactions demonstrated a need for greater cultural humility when designing related curricula for Global South learners.

In the current pandemic environment, these lessons should not be ignored: African countries are under pressure to enact a robust COVID-19 response, just as they have needed to, in the past, for tuberculosis, HIV/AIDS, cholera, malaria, leprosy, and many other infectious or epidemic diseases. However, without adequate attention to the design and delivery of related training efforts, both the health security and diplomacy efforts of the Global South will inevitably be limited, most particularly by not including local actors as integral and vital problem solvers. In turn, such limitations threaten the health security of more affluent countries; building international health security capacity is, undoubtedly, in the global interest.

## Data Availability

N/A
